# Wearable EMG Measurement Device Using Polyurethane Foam for Motion Artifact Suppression

**DOI:** 10.3390/s24102985

**Published:** 2024-05-08

**Authors:** Takuma Takagi, Naoto Tomita, Suguru Sato, Michitaka Yamamoto, Seiichi Takamatsu, Toshihiro Itoh

**Affiliations:** Department of Precision Engineering, Graduate School of Engineering, The University of Tokyo, 7-3-1, Hongo, Bunkyo-ku, Tokyo 113-8654, Japan; toonatamito@g.ecc.u-tokyo.ac.jp (N.T.); sugurusato-hem@g.ecc.u-tokyo.ac.jp (S.S.); takamatsu@pe.t.u-tokyo.ac.jp (S.T.); itoh@pe.t.u-tokyo.ac.jp (T.I.)

**Keywords:** wearable device, EMG measurement, polyurethane foam, motion artifact

## Abstract

We propose the use of a specially designed polyurethane foam with a plateau region in its mechanical characteristics—where stress remains nearly constant during deformation—between the electromyography (EMG) electrode and clothing to suppress motion artifacts in EMG measurement. Wearable EMG devices are receiving attention for monitoring muscle weakening due to aging. However, daily EMG measurement has been challenging due to motion artifacts caused by changes in the contact pressure between the bioelectrode and the skin. Therefore, this study aims to measure EMG signals in daily movement environments by controlling the contact pressure using polyurethane foam between the bioelectrode on the clothing and the skin. Through mechanical calculations and finite element method simulations of the polyurethane foam’s effect, we clarified that the characteristics of the polyurethane foam significantly influence contact pressure control and that the contact pressure is adjustable through the polyurethane foam thickness. The optimization of the design successfully controlled the contact pressure between the bioelectrode and skin from 1.0 kPa to 2.0 kPa, effectively suppressing the motion artifact in EMG measurement.

## 1. Introduction

In recent years, biometric wearable devices have garnered significant attention for visualizing human health conditions [1,2,3]. Numerous device types, such as heart rate monitoring devices [4,5], electrocardiogram (ECG) measurement devices [6,7], and electromyogram (EMG) measurement devices [8,9,10], have been developed. Among these, EMG measurement devices are essential for improving healthy life expectancy because they can monitor the magnitude of a force caused by muscles [11] and the loss of muscle mass due to aging [12,13], which should be continuously monitored and fed back into daily life. Furthermore, wearable EMG devices can realize noninvasive human–machine interfaces, proving effective for rehabilitative devices and prosthetics [14,15,16].

In bioelectrical measurements, such as ECG or EMG, bioelectrodes are placed in contact with the skin to measure the electrical signals generated during muscle and heart movements. For daily bioelectric measurement, two fundamental techniques are involved: developing long-life bioelectrodes and establishing a comfortable and reliable method for attaching bioelectrodes to human skin. Various types of long-life bioelectrodes for biomedical measurement have been developed, including ionic liquid gel-based electrodes [7,17], fiber electrodes [18,19], and conductive polymer electrodes [20,21]. In considering the bioelectrode attachment method, attaching the bioelectrode correctly to human skin to decrease the motion artifact caused by changes in contact is imperative. Several methods, such as attaching bioelectrodes to the skin directly by adhesive seals [22,23], tightening bioelectrodes by belt or band [24,25], or embedding bioelectrodes on or in clothing [26], have been suggested. While the method of embedding a bioelectrode in or on clothing is the most comfortable for daily measurement, it is challenging to maintain stable contact between the bioelectrode and skin. Previous studies have reported that the contact between the skin and bioelectrode—the contact pressure between the skin and bioelectrode—affects signal stability [27]. However, it is challenging to control the contact pressure between bioelectrodes and the skin; bioelectrodes embedded in or on clothing and skin may be out of contact during human movements, resulting in an error in EMG or ECG measurement [28,29].

To control the contact pressure, attempts have been made using polyurethane foam. For instance, a conductive polyurethane foam block was fabricated and attached to a shirt to stabilize contact with human skin [28], and silver-plated fibers were implanted into polyurethane foam to reduce motion artifacts during ECG measurement [30]. Although thick and soft polyurethane foams have been utilized to enhance ECG measurement stability by suppressing motion artifacts, the characteristics of polyurethane foam have not been effectively exploited, and design methods for using polyurethane foams are lacking. Polyurethane foam is a superelastic material with a plateau region in its mechanical characteristics, where deformation proceeds at an almost constant stress value. While the plateau region is thought to be effective in reducing motion artifacts, previous studies have not addressed its impact. Furthermore, insufficient discussion exists regarding the appropriate material and shape of the polyurethane foam to be utilized. This is due to the difficulty of establishing a simple design or calculation method for the effects of using nonlinear materials, such as polyurethane foam.

Therefore, this study investigated a structure placing polyurethane foam between clothing and bioelectrodes to create a wearable device for stable EMG measurement while examining key parameters in the design of the proposed structure with polyurethane foam. This study employed a theoretical model of material mechanics and revealed the importance of both the physical properties of the polyurethane foam and the thickness of the foam. Furthermore, based on the theoretical model, a finite element method (FEM) simulation was performed to investigate the effect of polyurethane foam thickness. Finally, the effect of the proposed structure and the importance of the optimal design were evaluated through actual device fabrication and EMG measurement.

## 2. Materials and Methods

### 2.1. Proposed Structure of Wearable EMG Measurement Device Using Polyurethane Form

Following a previous study, the effect of motion artifacts can be expressed by the following equation [31]:

(1) $$V_{ma}=2\left[∆V_{\mathrm{dc-offset}}+∆Z_{\mathrm{skin-electrode}}\left(\frac{\left(\frac{V_{signal}}{2}\right)+V_{\mathrm{dc-offset}}}{Z_{in}}+I_{bias}\right)\right]$$

where *V_dc-offset_* and *Z_skin-electrode_* are the offset voltage and impedance between the skin and electrodes, and *I_bias_* is the bias current. As Δ*V_dc-offset_* is caused by the change in skin thickness and is challenging to control, making *Z_skin-electrode_* stable to suppress the motion artifact is crucial. *Z_skin-electrode_* is known to be affected by the contact condition and pressure between bioelectrodes and the skin. Therefore, maintaining contact pressure is essential to suppress motion artifacts during bioelectric measurement.

**Figure 1:**
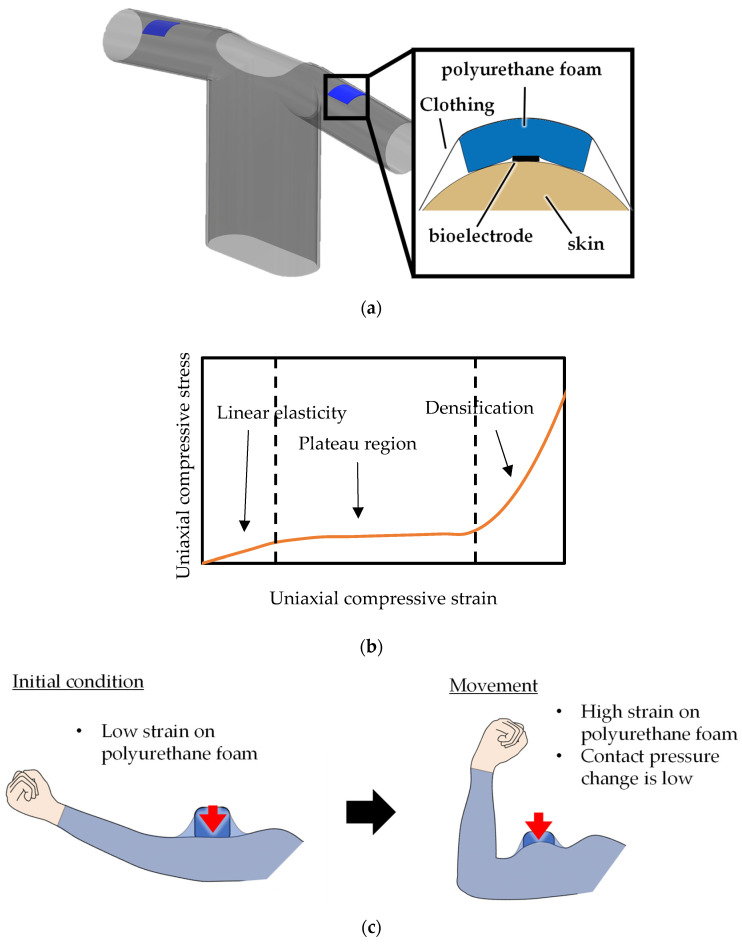
(**a**) The proposed structure of a wearable EMG measurement device using polyurethane foam. (**b**) The plateau region of the polyurethane foam. (**c**) The contact pressure stabilization mechanism using polyurethane foam. The red arrows in the figure show the direction of the force generated by the urethane foam and the clothes.

Figure 1a shows the proposed structure of a wearable EMG measurement device using polyurethane foam. The proposed structure comprises polyurethane foam between the clothing and bioelectrodes. The contact pressure between the bioelectrode and skin is controlled using a plateau region (Figure 1b), where the polyurethane foam deforms under almost constant stress. When the body’s diameter increases due to movement, such as arm bending, the tension acting on the fabric increases, causing heightened contact pressure in conventional structures without polyurethane foam. Conversely, polyurethane foam with plateau regions absorbs the body diameter increase in the proposed structure, as shown in Figure 1c. This absorption by polyurethane foam helps control the contact pressure, ensuring its consistency despite changes in arm diameter. This contact pressure stability reduces the change in *Z_skin-electrode_*, thereby reducing the motion artifacts and leading to high-precision EMG measurement.

**Figure 2:**
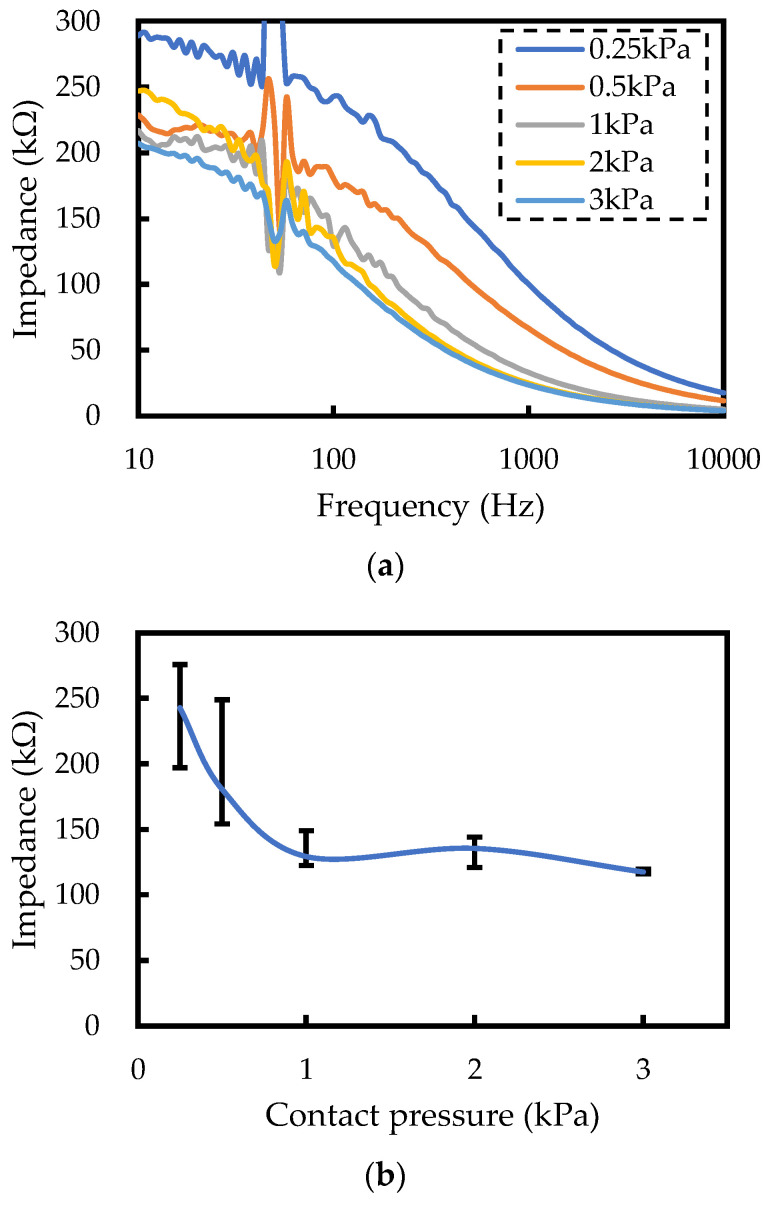
The experimental measurement results of the relationship between contact pressure and impedance. (**a**) The relationship between frequency and impedance at different contact pressures. (**b**) The relationship between contact pressure and impedance at a measurement of 100 Hz.

To obtain reliable EMG measurements, establishing the optimal contact pressure between the bioelectrode and the skin is crucial. Therefore, the relationship between contact pressure and impedance was evaluated. The bioelectrode reported in previous work [32] was prepared, and the contact pressure and impedance between the skin and electrode were measured using a load cell (MODEL-3005, Aicoh Engineering, Aichi, Japan) and an inductance, capacitance, and resistance (LCR) meter (M3353, Hioki Electric, Nagano, Japan). Figure 2 shows the measurement results. Figure 2a shows that the impedance values decreased with increasing contact pressure, except for around 50 Hz, where power supply noise interfered with accurate measurements. Figure 2b shows the impedance value at 100 Hz, showing significant changes in values when the contact pressure is below 1.0 kPa, while impedance remains stable when the contact pressure is above 1.0 kPa. Notably, contact pressure exceeding 2.0 kPa causes discomfort and constriction in the body [33]. Therefore, clothing-type devices should be designed to ensure that the contact pressure does not exceed 2.0 kPa. That is, the optimal range of the contact pressure for EMG measurement results is 1.0–2.0 kPa.

### 2.2. Theoretical Calculation of Contact Pressure with or without Using Polyurethane Form

We evaluated the contact pressure between bioelectrodes and the skin theoretically in cases with and without polyurethane foam. First, without polyurethane foam, the contact pressure can be calculated using Kirk’s equation [34]:

(2) $$P=\frac{T_{h}}{r_{h}}+\frac{T_{v}}{r_{v}}$$

where *T_h_* and *T_v_* are the warp and weft yarn tension [N/m], and *r_h_* and *r_v_* are the radius of curvature of the warp and weft yarns [m]. The tension *T* can be calculated from the Young’s modulus *E_cloth_* [Pa] of the garment, the thickness *t* [m], and the amount of elongation *ε* of the garment, as follows:

(3) $$T=E_{cloth}t\varepsilon$$

Notably, each value should be calculated or estimated on a measured value basis.

**Figure 3:**
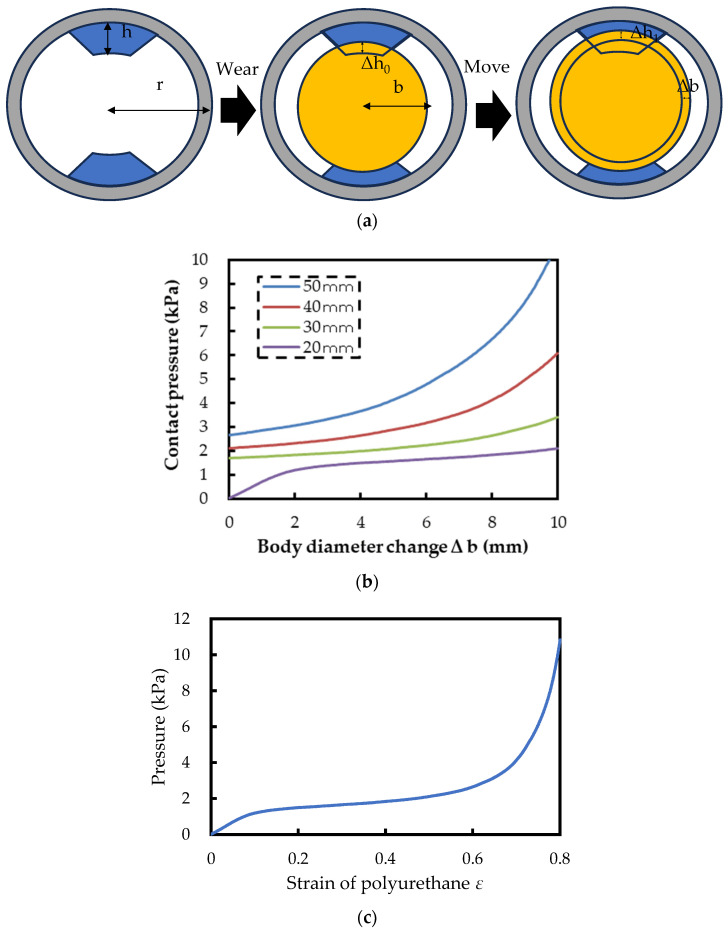
The theoretical calculation model and results. (**a**) The model used for the theoretical calculation. (**b**) The calculation results of the described conditions. (**c**) The characteristics of the polyurethane foam used.

Next, we considered the proposed structure using polyurethane foam. The theoretical calculation model considered this time is shown in Figure 3a. When preparing the proposed structure with the radius curvature of the wear *r* and the thickness of the polyurethane foam *h*, the change in each part when a person wears the proposed structured wear is considered as follows:

(4) $$r=b+h-\Delta h_{0}$$

where *b* is the body’s radius, and Δ*h*_0_ is the change in the polyurethane foam’s thickness. In the above state, the contact pressure is equal to the pressure that deforms the polyurethane foam, so the contact pressure can be represented using the function *E_ure_*(*ε*), which shows the relationship between the strain in polyurethane and the pressure produced by the strain as follows:

(5) $$P=E_{ure}(\frac{\Delta h_{0}}{h})$$

Notably, the function *E_ure_*(*ε*) must be determined separately to calculate the actual pressure. In most cases, actual measurement is required.

When the diameter of the body changes from *b* to *b* + Δ*b* by movement, the following equation is formed using the change in the polyurethane foam’s thickness Δ*h*_1_:

(6) $$r=b+\Delta b+h-\Delta h_{0}-\Delta h_{1}$$

Furthermore, the contact pressure at that time can be written as follows:

(7) $$P=E_{ure}(\frac{\Delta h_{0}+\Delta h_{1}}{h})$$

From Equations (6) and (7), the contact pressure can be converted as follows:

(8) $$P=E_{ure}(\frac{\left(h+b\right)-r+\Delta b}{h})$$

This means that the contact pressure is determined by the physical properties of the polyurethane foam used, the diameter of the clothing, which corresponds to the size of the clothing, the thickness of the polyurethane foam used, and the diameter of the body. Considering that the clothing size and the body’s diameter are not parameters that can be designed, this formula basically means that the thickness *h* of the polyurethane foam can control the contact pressure.

Figure 3b shows the relationship between body diameter and contact pressure. The thickness of the polyurethane foam varies from 20 to 50 mm in 10 mm increments, and Figure 3c shows the characteristics of the polyurethane foam used. The polyurethane foam used is the 4979874852328 provided by Yahata Neji Co., Ltd. (Saitama, Japan). The clothing radius was 60 mm, and the body radius changed from 40 mm to 50 mm with movement. The thicker the polyurethane foam, the higher the initial contact pressure and the smaller the slope of the pressure change during body movement. However, if the thickness of the polyurethane foam becomes too large, the range of the stable contact pressure area may be reduced.

Notably, this calculation assumes the use of soft polyurethane foam that can effectively absorb any changes in the body, and the diameter of the clothing remains unchanged. If softer clothes are used and their diameter changes, we need to represent “*r*” as “*r +* Δ*r*_0_” in Formula (4) and consider the later section’s change in “*r*”. However, given that it can complicate the calculation, we have described the calculation in Supplementary Materials S1 for such cases. As the amount of strain on the clothing is linked to the change in the polyurethane foam’s thickness, it is challenging to give a general explanation. However, we speculated that an approximate determination of clothing softness could be made by considering the clothing material, the polyurethane foam, and the balance of the respective forces. The discussion is provided in Supplementary Materials S2.

### 2.3. Simulation Method

**Figure 4:**
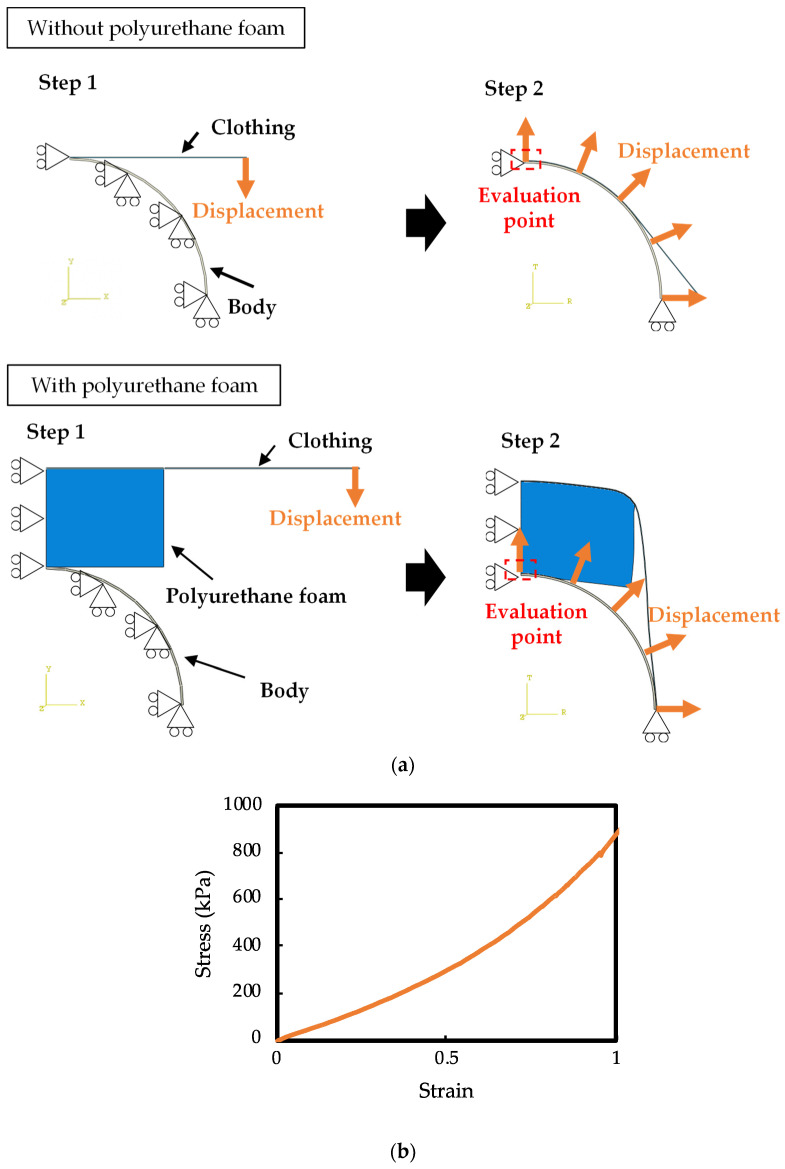
The FEM simulation model. (**a**) The FEM simulation model with and without polyurethane foam. The red point shows the evaluation point of the contact pressure. (**b**) The characteristics of the clothing used.

The simulation was performed using the FEM software Abaqus 2022. A two-dimensional 1/4 model of the cross-section was used by considering symmetry. The model used for the simulation is shown in Figure 4a. A human body, modeled as a cylindrical rigid body along with polyurethane foam and clothing, was placed. In Step 1, the human body was dressed by applying a y-directional displacement to the right end of the clothing. The boundary condition of complete fixation was imposed on the human body, and restraints were imposed in the x-direction on the left end of the clothing and polyurethane foam. In Step 2, the human body was moved in the r-direction in a cylindrical coordinate system to represent changes in the shape of the human body due to movement. In this simulation, assuming that the target of EMG measurement is a human arm, the radius of the human body *r* was varied from 35 mm to 55 mm, and the contact pressure between the wear and the skin was calculated for the area enclosed by the red frame in Figure 4a. The coefficient of friction between the garment and the human body was set at 0.8 [35]. Notably, the length of the cloth is 1/4 of the actual length of the entire circumference of the cloth, and the polyurethane foam’s width is 1/2 of the actual width of the polyurethane foam because the calculations were performed using a two-dimensional 1/4 model of the cross-section.

Using FEM simulation, the comparison between with and without polyurethane foam and the effect of polyurethane thickness were evaluated. This is because the thickness of the polyurethane foam is expected to significantly affect the results based on theoretical calculations. The actual measurement results for the clothing and polyurethane foam were used in the simulation. The measurement result of clothing is shown in Figure 4b while that of polyurethane foam remains the same as that used in the previous theoretical calculation. In the simulation of the polyurethane thickness evaluation, the polyurethane thickness was changed from 15 to 30 mm. The parameters of the FEM simulation model used for evaluating the effect of polyurethane foam are summarized in Table 1. The thickness of the polyurethane foam was changed from 15 mm to 30 mm to investigate the thickness of the polyurethane foam. The other parameters are the same as in Table 1.

### 2.4. Experimental Method

To experimentally evaluate the contact pressure, measurements were taken with and without polyurethane foam and at different thicknesses ranging from 10 mm to 50 mm. The cylindrical models of the upper arm with different diameters (40, 45, and 50 mm radius) were fabricated using a 3D printer (Raise 3D E2, Raise 3D Technologies Inc., Irvine, CA, USA), and the contact pressure was measured with an air-pack-type contact surface pressure measuring system (AMI3037-10-SW, AMI Techno, Tokyo, Japan) for each condition. The width of the polyurethane foam was fixed at 50 mm.

The previously reported ionic liquid gel-type bioelectrode [32] was fabricated and used for the EMG measurement experiment. The fabricated bioelectrode and polyurethane foam were attached to the bicep area of the compression wear (KOHNAN SHOJI Co., Ltd., Osaka, Japan, FW19-OZ10) by adhesion tape. The image of the ionic liquid gel-type bioelectrode, wear, and polyurethane foam used is shown in Supplementary Materials S3. The polyurethane foam’s thickness was set to 0 mm (without polyurethane foam), 10 mm, 25 mm, and 50 mm. The bicep muscle was contracted three times during a one-minute period, and the signal was measured during these contractions.

The amplifier chip (Intan Technologies, Los Angeles, CA, USA, RHD 32-Channel Recording Headstages) and interface board (Intan, RHD USB Interface Board) were used for EMG measurement. The sampling rate was set to 20 kHz, and a 50 Hz notch filter was applied to eliminate the power supply noise. Notably, commercially available wet electrodes (BlueSensor P, Ambu, Ballerup, Denmark) were used as ground and reference electrodes.

The signal-to-noise ratio (SNR) was calculated for the measured results using the following formula:

(9) $$SNR=10\log_{10}\frac{Var(s(t))}{Var(n\left(t\right))}$$

where *s*(*t*) is the EMG signal after filtering, and *n*(*t*) is the noise. Var refers to the variance of each. The noise *n*(*t*) was obtained from the measured raw data *x*(*t*) and the filtered EMG signal *s*(*t*) using the following formula:

(10) $$n\left(t\right)=x\left(t\right)-s(t)$$

The filter was a fourth-order Butterworth filter with a passband of 20–450 Hz to remove motion artifacts and high-frequency noise.

## 3. Results

### 3.1. Simulation Results

**Figure 5:**
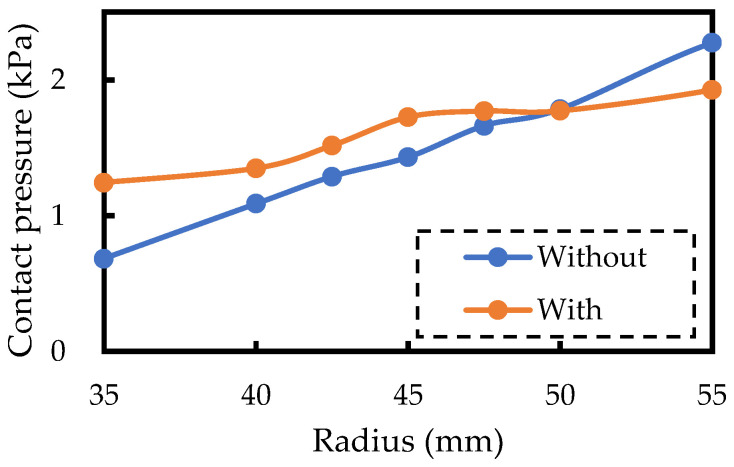
The simulation results of the relationship between the arm radius and contact pressure with and without polyurethane foam.

Figure 5 shows the simulation results of the relationship between the arm radius and contact pressure with and without polyurethane foam. When the radius of the human body changes from 35 mm to 55 mm, the contact pressure without polyurethane foam changes from 0.7 kPa to 2.3 kPa, a difference of 1.6 kPa, while with polyurethane foam, the contact pressure changes from 1.2 kPa to 2.0 kPa, a difference of only 0.8 kPa. This indicates that the use of urethane foam stabilizes the contact pressure.

**Figure 6:**
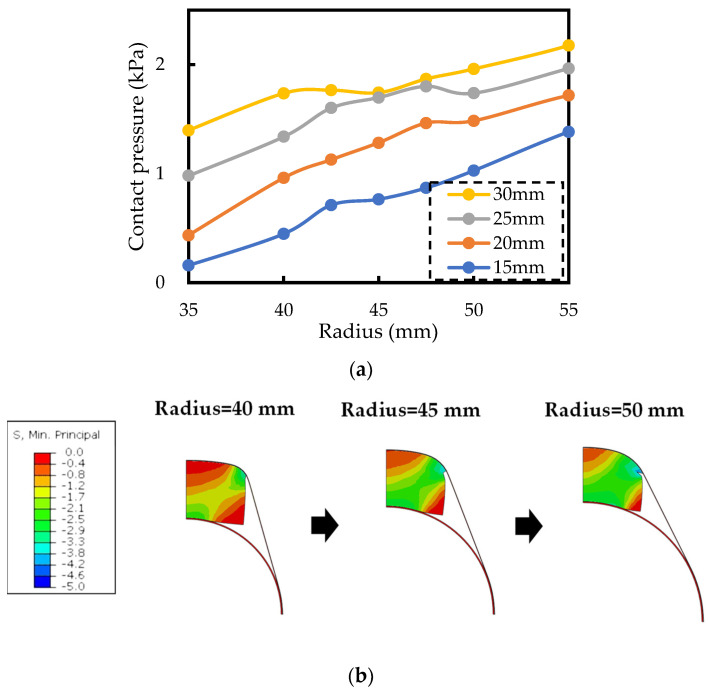
The simulation results of the different polyurethane foam thicknesses. (**a**) The simulation result of the influence of polyurethane thickness on contact pressure by each radius. (**b**) The simulation result of the minimum stress distribution at a polyurethane foam thickness of 25 mm.

Figure 6a shows the simulation results of the relationship between the radius of the human body and the contact pressure when the polyurethane foam thickness is changed. Figure 6b shows an example of the simulation result, demonstrating the minimum stress distribution at a polyurethane foam thickness of 25 mm. Figure 6a shows that as the polyurethane foam thickness increases, the overall pressure also increases, but the slope of the curve becomes more gradual when the radius of the human body changes. This result aligns with the theoretical calculation, and the higher overall pressure is likely due to the increase in the initial pressure.

### 3.2. Contact Pressure Measurement Experiment Results

**Figure 7:**
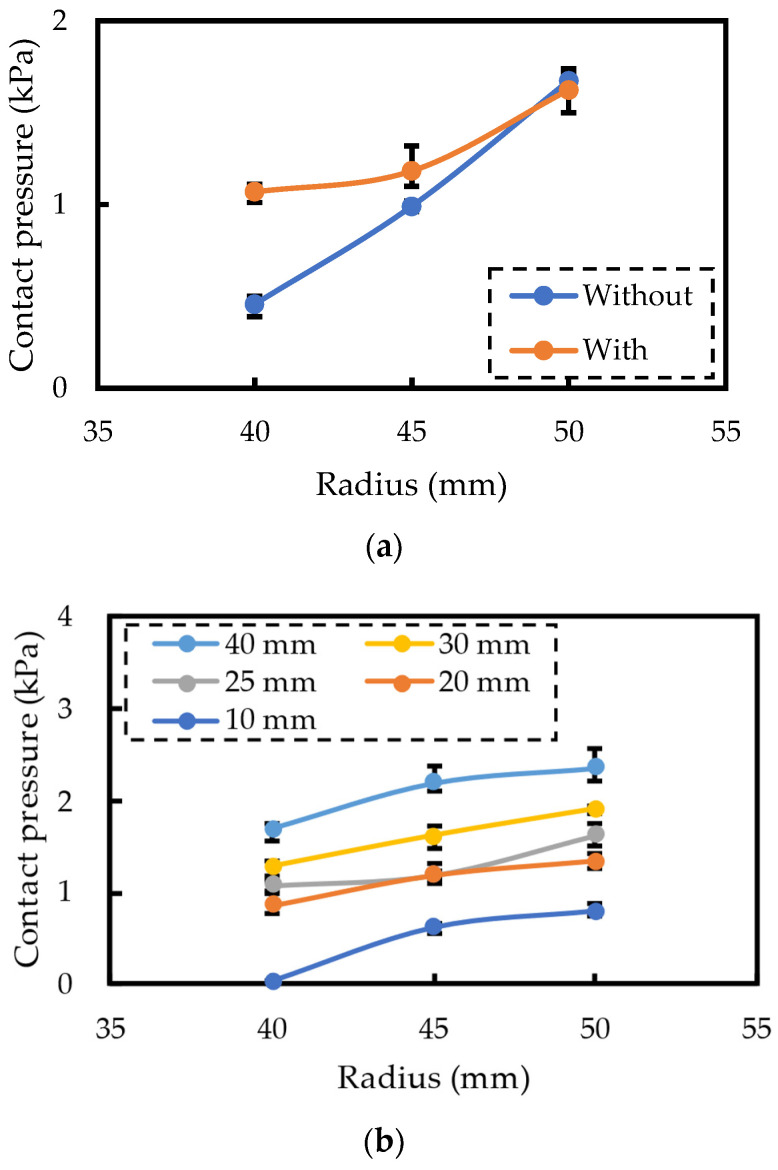
Experimental results of contact pressure measurement. (**a**) Comparison with and without polyurethane foam. (**b**) Influence of polyurethane foam thickness.

Figure 7a shows the experimental measurement results of the contact pressure with and without polyurethane foam. Without polyurethane foam, the contact pressure increased by 1.2 kPa (240%) from 0.5 kPa to 1.7 kPa, while with polyurethane foam, the contact pressure increased only by 0.6 kPa (45%) from 1.1 kPa to 1.6 kPa. This indicates that the proposed structure stabilized the contact pressure.

Figure 7b shows the experimental measurement result of changing the polyurethane foam thickness. It indicates that the overall pressure increases with increasing thickness, which aligns with theoretical calculations and FEM simulation results. In particular, the slope of change with a thickness exceeding 20 mm becomes smaller than that with a thickness of 10 mm. Although the results are more varied than the simulation results owing to experimental factors, the simulation results are considered to be approximately correct. It is evident that by designing the correct thickness, the contact pressure can be successfully controlled within the range of 1–2 kPa.

### 3.3. EMG Measurement Experiment Results

**Figure 8:**
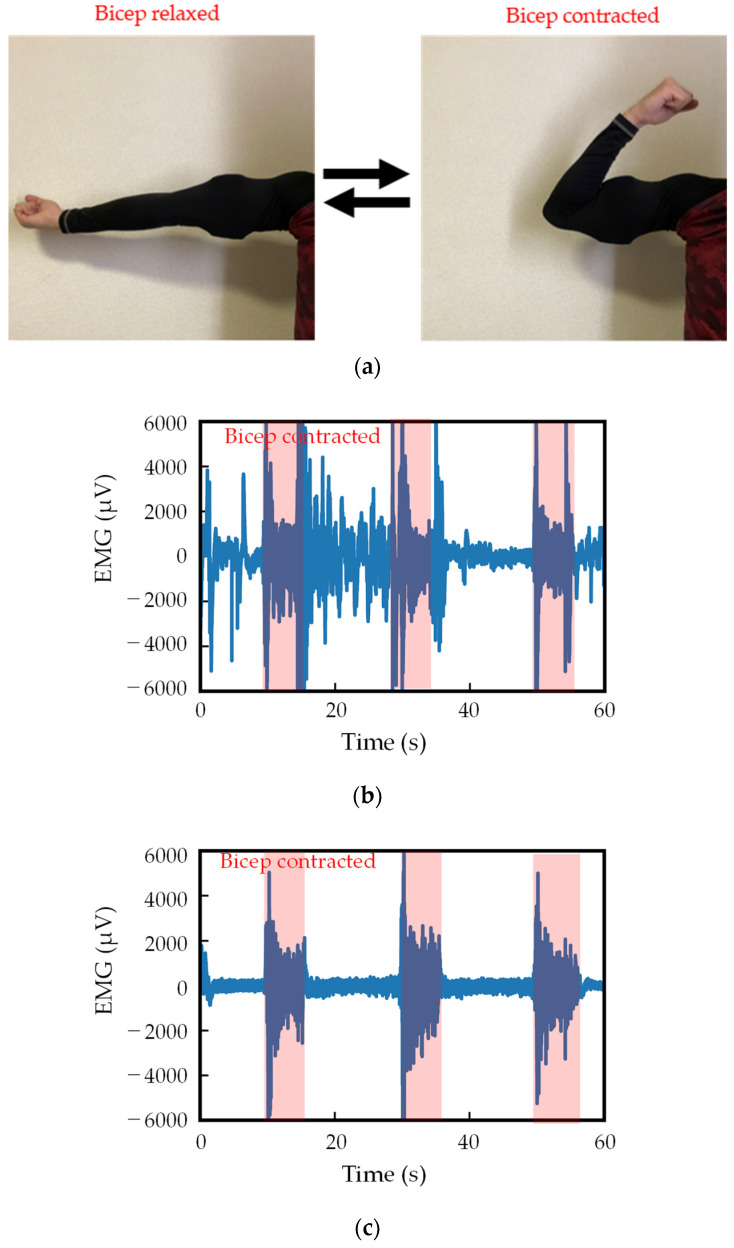
EMG measurement results with and without polyurethane foam. The red-colored parts are the bicep contracted area. (**a**) Measurement images of bicep relaxed and contracted. (**b**) Without polyurethane foam. (**c**) With polyurethane foam.

The EMG measurement results with or without polyurethane foam are shown in Figure 8. The measurement images of a bicep relaxed and contracted are shown in Figure 8a, and the measured EMG signals without and with polyurethane foam are shown in Figure 8b,c, respectively. The signal processing methodology applied in Figure 8b,c involved using a notch filter during measurement, with no postsignal filtering implemented. Figure 8b,c show that with polyurethane foam allows for EMG measurement with reduced noise, whereas without polyurethane foam results in a significant amount of noise. The SNR improved from −4.4 dB without polyurethane foam to 1.75 dB with polyurethane foam.

**Figure 9:**
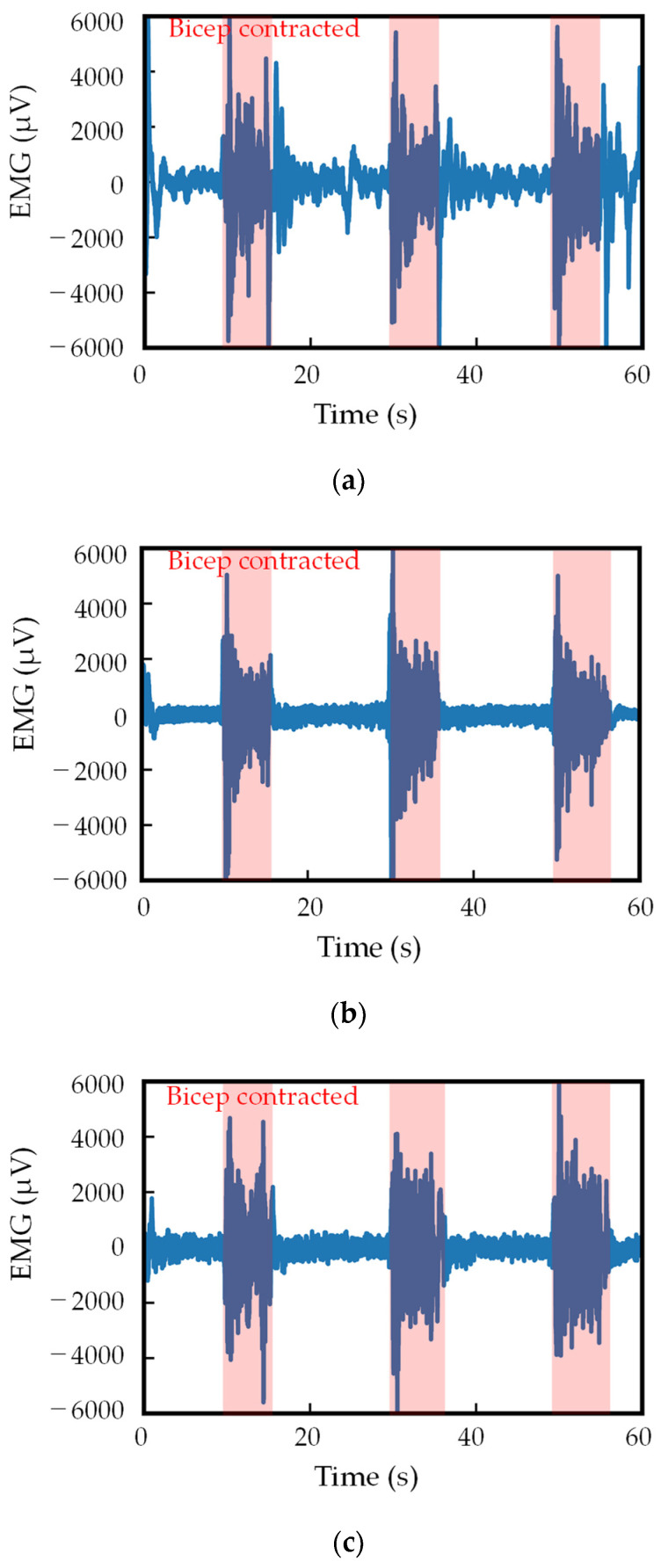
EMG measurement results of different polyurethane foam thicknesses: (**a**) 10 mm, (**b**) 25 mm, and (**c**) 50 mm.

**Figure 10:**
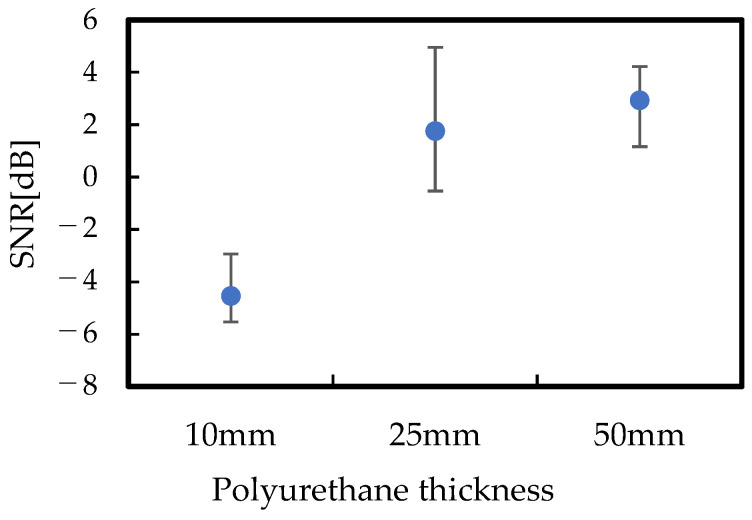
The SNR calculation results of different polyurethane thicknesses.

The EMG measurement results for different polyurethane foam’s thicknesses are shown in Figure 9, and the SNR calculation results for that case are shown in Figure 10. When the polyurethane foam thickness is 10 mm, a large amount of noise exists. This is because the contact pressure was small, and the motion artifact reduction effect of the polyurethane was insufficient, resulting in significant noise. However, increasing the polyurethane foam thickness enabled the acquisition of increasingly precise myoelectric signals, resulting in an improved SNR. Notably, while the contact pressure will be stable, an excessive thickness of the polyurethane foam can lead to discomfort for humans. Therefore, proper design is essential.

Furthermore, when calculating the SNR based on the ratio of the variance between data within the contraction region and those within the noncontraction region, the resulting SNR values are as follows: 10.5 dB without the use of polyurethane foam, 17.7 dB with a polyurethane foam thickness of 10 mm, 20.0 dB with a thickness of 25 mm, and 20.3 dB with a thickness of 50 mm. These results further support the effectiveness of using properly designed polyurethane foam.

## 4. Conclusions

This study proposed a method to reduce motion artifacts during EMG measurement using polyurethane foam, established a design method based on material mechanics and FEM simulation, and experimentally demonstrated its effectiveness.

Through mechanical calculations, it was clarified that the characteristics of the polyurethane foam significantly affect the control of the contact pressure and that the contact pressure can be adjusted by the polyurethane foam thickness. We designed the EMG measurement wear through FEM simulation and controlled the contact pressure between 1.0 kPa and 2.0 kPa, ensuring stable impedance between the bioelectrode and the skin. The designed EMG measurement wear successfully measured EMG signals, and the SNR was improved from −4.4 dB without polyurethane foam to 1.75 dB with polyurethane foam. The proposed method of using polyurethane foam and its design method are expected to be beneficial for fabricating wearable biosensing devices.

## Figures and Tables

**Table 1:** The parameters of the FEM simulation model.

Model	Length of Clothing	Width of Polyurethane Foam	Thickness of Polyurethane Foam
Without polyurethane foam	45	-	-
With polyurethane foam	80	30	25 mm or 15–30 mm

## Data Availability

All the data have been included in the study.

## Supplementary Materials

The following supporting information can be downloaded at: https://www.mdpi.com/article/10.3390/s24102985/s1. S1: The theoretical calculation for soft clothing and changing clothing diameter; S2: The relationship between fabric tension and urethane balance based on material mechanics calculations. S3: The images of the ionic liquid gel-type bioelectrode, wear, and polyurethane foam used.
