# Supplementary material for: Wearable EMG Measurement Device Using Polyurethane Foam for Motion Artifact Suppression
Source: Sensors (Basel). 2024 May 8;24(10):2985. doi: 10.3390/s24102985 (PMC11124951; doi:10.3390/s24102985)
Supplement: Supplementary file 1 [file sensors-24-02985-s001.zip › sensors-2929858-supplementary.pdf]

Supplementary Materials S1:
Theoretical calculation for soft cloth and changing cloth diameter

Consider the case where the cloth is soft and the radius of the clothing changes with human motion. By considering the proposed structure with radius curvature of the wear r and the polyurethane foam thickness h, the change of each part when a person wears the proposed structured wear is considered as follows:

$$r + \Delta r_0 = b + h - \Delta h_0 \qquad \text{(S1-1)}$$

where b is the body's radius, Δh_0 is the change in the polyurethane foam thickness, and Δr_0 is the wear's changed radius.

In the above state, the contact pressure is equal to the pressure that deforms the polyurethane foam, so the contact pressure can be represented using the function $E_{ure}(\varepsilon)$, which shows the relationship between the strain in the polyurethane and the pressure produced by the strain as follows:

$$P = E_{ure}\left(\frac{\Delta h_0}{h}\right) \qquad \text{(S1-2)}$$

Notably, the function $E_{ure}(\varepsilon)$ must be determined separately to calculate the actual pressure. In most cases, actual measurement is required.

When the body's diameter changes from b to $b+\Delta b$ by the movement, the next equation holds using the change of the polyurethane foam thickness Δh_1 and the change of the wear radius Δr_1:

$$r + \Delta r_0 + \Delta r_1 = b + \Delta b + h - \Delta h_0 - \Delta h_1 \qquad \text{(S1-3)}$$

Furthermore, the contact pressure at that time can be written as follows:

$$P = E_{ure}\left(\frac{\Delta h_0 + \Delta h_1}{h}\right) \qquad \text{(S1-4)}$$

From equations (3) and (4), the contact pressure can be converted as follows:

$$P = E_{ure}\left(\frac{(h+b) - r - \Delta r_0 - \Delta r_1 + \Delta b}{h}\right) \qquad \text{(S1-5)}$$

As the amount of strain on the clothing is linked to the change in the polyurethane foam thickness, it is challenging to give a general explanation. However, the approximate change trend is that lengthening the diameter of the clothing, which corresponds to wearing flexible clothing or soft clothing material, leads to a reduction in Δh_1, thereby decreasing the contact pressure. We considered that a rough assessment of whether the clothing is softer could be made by considering the material of the clothing, the polyurethane foam, and the balance of the respective forces. The discussion is provided in Supplementary Materials S2

Supplementary Materials S2:
Relationship between fabric tension and urethane balance based on material mechanics calculations

In this appendix, the effect of the hardness of clothing and polyurethane foam is considered. First, in the case without polyurethane foam, the contact pressure can be calculated by following Kirk's Equation [S2-1]:

$$P = \frac{T_h}{r_h} + \frac{T_v}{r_v} \qquad \text{(S2-1)}$$

where T_h and T_v are the warp and weft yarn tension [N/m] and r_h and r_v are the radius of curvature of warp and weft yarns [m]. Notably, the radius of curvature in the vertical direction r_v can be taken to be large enough to be ignored based on the human body structure. When considering putting the polyurethane foam with thickness h, the radius of the clothing r_h can be expressed as follows by incorporating the body radius b:

$$r_h = b + h \qquad \text{(S2-2)}$$

here, the thickness of the clothing t was ignored because it is small compared to body diameter and polyurethane foam thickness.

The tension T can be calculated from Young's modulus E_{cloth} [Pa] of the clothing, the thickness of clothing t [m], and the strain of the clothing ε of the garment as follows:

$$T_{hinitial} = E_{cloth} t \varepsilon \qquad \text{(S2-3)}$$

When considering the change in the body diameter Δb and polyurethane thickness Δh, and assuming that each is deformed while maintaining its cylindrical shape, the relationship between them is as follows:

$$\varepsilon = \frac{\Delta b + \Delta h}{b + h} \qquad \text{(S2-4)}$$

Thus, the contact pressure on polyurethane foam can be written as follows:

$$P = \frac{E_{cloth} t(\Delta b + \Delta h)}{(b+h)^2} \qquad \text{(S2-5)}$$

Notably, it was assumed to be the region where $b + h + \Delta b + \Delta h \approx b + h$.

Conversely, by considering the relationship between polyurethane strain and pressure, the relationship can be written as follows because Δh is lower than 0:

$$P = E_{ure} \frac{-\Delta h}{h} \qquad \text{(S2-6)}$$

here, E_{ure} is the young's module of the polyurethane foam. Notably, while the definition in the main text is presented as a function, it is defined as a constant here. By considering Formulas (5) and (6), the relationship can be expressed as follows:

$$P = \frac{E_{cloth} \cdot t \cdot \Delta b}{(b+h)^2 + \frac{E_{cloth}}{E_{ure}} \cdot t \cdot h} = \frac{E_{ure} \cdot t \cdot \Delta b}{\frac{E_{ure}}{E_{Cloth}}(b+h)^2 + t \cdot h} \qquad \text{(S2-7)}$$

This means that the case can be divided based on the ratio of E_{cloth} to E_{ure} as follows:

① $E_{ur} \gg E_{cloth}$: $P = \frac{E_{cloth} \cdot t \cdot \Delta b}{(b+h)^2}$

② $E_{ur} \ll E_{cloth}$: $P = \frac{E_{ure} \cdot \Delta b}{h}$

Notably, in this case, the relationship between Δb and Δh is as follows:

$$\Delta h = \frac{E_{ure} \cdot h \cdot t \cdot \Delta b}{E_{cloth} \cdot h \cdot t + E_{ure} \cdot (b+h)^2} \qquad \text{(S2-8)}$$

and $\Delta h \fallingdotseq \Delta b$ because $E_{ure} \ll E_{cloth}$; it can be written as follows:

$$P = \frac{E_{ure} \cdot \Delta h}{h} \qquad \text{(S2-9)}$$

This indicates that, in this case, only the deformation of the sponge and the resulting pressure need to be considered.

③ $E_{ur} \fallingdotseq E_{cloth}$: The above formula (S2-7).

[S2-1] Kirk Jr., W.; Ibrahim, S. Fundamental relationship of fabric extensibility to anthropometric requirements and garment performance. Text. Res. J. 1966, 36, 37-47.

Supplementary Materials S3:
The images of the ionic liquid gel type bioelectrode, wear, and polyurethane foam used are provided

The image of the ionic liquid gel type bioelectrode, wear, and polyurethane foam used is shown in Figure S3. For further details regarding the fabrication process, please refer to a previous study [S3-1].

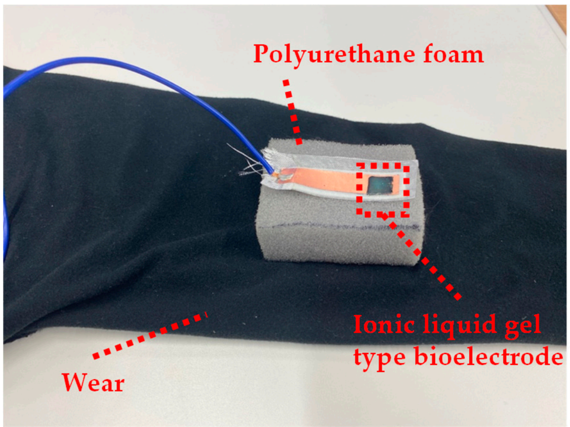

Figure S3. The image of the used ionic liquid gel type bioelectrode, along with the polyurethane foam and the wear used in the experiment.

[S3-1] Tomita, N.; Takamatsu, S.; Itoh, T., Fabrication of an E-Textile Bioelectrode Array with Screen-Printed Wiring and an Ionic Liquid Gel toward Cutaneous Whole-Body Electromyography. *IEEE Access* **2023**, 11, 68421-68427.
